# Conifers and non-native tree species shift trophic niches of generalist arthropod predators in Central European beech forests

**DOI:** 10.1186/s12862-023-02105-1

**Published:** 2023-02-03

**Authors:** Wildermuth Benjamin, Fardiansah Riko, Matevski Dragan, Lu Jing-Zhong, Kriegel Peter, Scheu Stefan, Schuldt Andreas

**Affiliations:** 1 Forest Nature Conservation, University of Göttingen, Büsgenweg 3, 37077 Göttingen, Germany; 2 Johann-Friedrich-Blumenbach Institute of Zoology and Anthropology, University of Göttingen, Untere Karspüle 2, 37077 Göttingen, Germany; 3 Field Station Fabrikschleichach, Department of Animal Ecology and Tropical Biology (Zoology III), University of Würzburg, Glashüttenstraße 5, 96181 Rauhenebrach, Germany; 4 Centre of Biodiversity and Sustainable Land Use, University of Göttingen, Büsgenweg 1, 37077 Göttingen, Germany

**Keywords:** Araneae, Biodiversity, Carabidae, Ecosystem functioning, Stable isotopes

## Abstract

**Background:**

Functional diversity is vital for forest ecosystem resilience in times of climate-induced forest diebacks. Admixing drought resistant non-native Douglas fir, as a partial replacement of climate-sensitive Norway spruce, to native beech forests in Europe appears promising for forest management, but possible consequences for associated biota and ecosystem functioning are poorly understood. To better link forest management and functional diversity of associated biota, we investigated the trophic niches (∆^13^C, ∆^15^N) of epigeic generalist predators (spiders and ground beetles) in mixed and pure stands of European beech, Norway spruce and non-native Douglas fir in north-west Germany. We assessed the multidimensional niche structure of arthropod predator communities using community-based isotopic metrics.

**Results:**

Whilst arthropod ∆^13^C differed most between beech (high ∆^13^C) and coniferous stands (low ∆^13^C), ∆^15^N was lowest in non-native Douglas fir. Tree mixtures mitigated these effects. Further, conifers increased isotopic ranges and isotopic richness, which is linked to higher canopy openness and herb complexity. Isotopic divergence of ground beetles decreased with Douglas fir presence, and isotopic evenness of spiders in Douglas fir stands was lower in loamy sites with higher precipitation than in sandy, drier sites.

**Conclusions:**

We conclude that tree species and particularly non-native trees alter the trophic niche structure of generalist arthropod predators. Resource use and feeding niche breadth in non-native Douglas fir and native spruce differed significantly from native beech, with more decomposer-fueled and narrower feeding niches in beech stands (∆^13^C, isotopic ranges and richness). Arthropod predators in non-native Douglas fir, however, had shorter (∆^15^N) and simplified (isotopic divergence) food chains compared to native forest stands; especially under beneficial abiotic conditions (isotopic evenness). These findings indicate potential adverse effects of Douglas fir on functional diversity of generalist arthropod predators. As tree mixtures mitigated differences between beech and conifers, mixed stands including (non-native) conifers constitute a promising compromise between economic and conservational interests.

**Supplementary Information:**

The online version contains supplementary material available at 10.1186/s12862-023-02105-1.

## Background

Biodiversity can stabilize resource use in ecosystems over time [1]. Therefore, biodiversity is an important indicator of ecosystem resilience and functioning of forests [2]. Tree species mixtures are known to show higher adaptability to changing environmental conditions than monocultures [3, 4]. In Central Europe, nonetheless, monocultures of the fast-growing Norway spruce (*Picea abies* (L.) H. Karst.) still constitute the most important timber supply, but detrimental effects of recent droughts on these monocultures have emphasized the need for new management strategies [5]. Planting mixtures of broadleaved and coniferous trees or admixing non-native trees to native stands is expected to increase forest resilience and productivity [6–8]. In particular, admixing drought resistant non-native Douglas fir (*Pseudotsuga menziesii* (Mirbel) Franco) to native European beech (*Fagus sylvatica* L.) forests is considered a promising approach for Central European forests [7]. However, the ecological consequences of planting such mixtures, including non-native and potentially invasive species like Douglas fir, are poorly understood and a possible threat to local biodiversity and ecosystem functioning [9, 10].

Arthropods are a key element of biodiversity, providing crucial functions such as decomposition, predation and herbivory, and thus are a good proxy to evaluate forest integrity and new forest management strategies [11, 12]. In temperate forests, densities of host-specific arthropod pests are lower in mixtures of broadleaved trees with conifers [13, 14], whereas no consistent effects of broadleaved-conifer mixtures on arthropod predator abundance and diversity have been found [15–17]. Admixing non-native Douglas fir to Central European forests showed ambiguous effects on forest arthropod communities [18]. Douglas fir promoted ground beetles compared to Norway spruce and European beech [19], but in young stands, Douglas fir reduced ground-dwelling spiders compared to native tree species [20]. Overall, Douglas fir harbored a different arthropod community than native tree species [21–23]. Despite these contrasting results, arthropod predators generally respond positively to structural stand properties, such as canopy openness and ground vegetation [17, 19] and tree species mixtures were found to mitigate the effects of single species [19, 24].

Although the above-mentioned studies considered some aspects of functional arthropod community structure, such as trophic guilds, they were mainly taxonomically oriented. Taxonomic diversity, however, provides no clear picture of the functional diversity and structure of arthropod communities. It needs exploration of functional community composition to consider the occupancy of ecological niches and thus, to draw conclusions on ecosystem functioning [25, 26]. Many important forest arthropod functions, such as predation, are based on trophic interactions, making feeding niches of arthropods a particularly important proxy for functional structure and diversity [27]. Generally, trophic niche breadth determines the likelihood of a species to cope with disturbances [28, 29] and in turn, trophic complexity promotes ecosystem functioning [30]. In this context, predators can be regarded as top-down control agents, including implications on the pest control in forests [31, 32]. In the ground stratum, spiders and ground beetles are major groups of generalist arthropod predators, which indirectly regulate herbivory and decomposition and are sensitive to habitat change [17, 33, 34]. This makes feeding niche structure of spiders and ground beetles an interesting and important parameter when evaluating sustainable forests management strategies.

Natural abundances of stable isotopes provide insight into trophic interactions and channels of energy. Isotope ratios of ^13^C/^12^C (δ^13^C) and ^15^N/^14^N (δ^15^N) are commonly used to assess animal trophic positions and dietary sources [35]. Whilst δ^13^C changes little over trophic levels but differs between carbon sources, enrichment of ^15^N in animal tissue relative to the food source provides information on the trophic level [35, 36]. Dual stable isotope analysis of animal δ^13^C and δ^15^N therefore allows the definition of a two-dimensional trophic niche, including the origin of food sources (δ^13^C) and the trophic position (δ^15^N) in a food web [35, 37]. Previous studies on interrelations between land use or forest type and isotopic signals of arthropods mostly focused on low taxonomic levels and their basic differences in δ^13^C and δ^15^N [38–40]. For the few studies that compared mean arthropod δ^13^C and δ^15^N across forest types, the results suggested that isotopic niches were little affected by forest types [41–43], whereas study region had a stronger influence [43]. Slightly lowered δ^13^C values (detrital shift) of arthropod consumers in coniferous stands compared to broadleaved stands were either assigned to restricted access to root-derived resources with high δ^13^C signatures in coniferous stands [39, 41, 42] or increased microbial activity, and thus higher δ^13^C in deciduous stands [44]. However, trophic niches are multidimensional and the variability of isotopic signals should be considered to determine the community-wide trophic niche structure (e.g. as quantified by variability measures of isotopic richness, divergence and evenness; [28, 36, 37]). Further, integrating the relative biomass of species within the community, accounts for dominant trophic structures more accurately compared to unweighted approaches [28, 45]. These recent methodological developments enable highly resolved analyses of trophic community structures, but are yet to be implemented in forest management research. All of the afore-mentioned studies lack integration of relative arthropod species biomass and do not provide multidimensional isotopic metrics of trophic niche structure. Furthermore, we have no information on how trophic niches of above-ground arthropods are affected by introducing non-native tree species, such as Douglas fir. Only one study considered mixtures of non-native Douglas fir with native Central European forests, and found similar trophic niches in oribatid species across forest types [47]. These research gaps might explain why differences in arthropod community composition between (non-native) stand types were not reflected by differences in trophic niche structure.

Our study addresses these knowledge gaps by analyzing trophic niches (δ^13^C and δ^15^N) of generalist predators [spiders (Araneae) and ground beetles (Carabidae)] in broadleaf-conifer mixtures and pure stands of native European beech, native Norway spruce and non-native Douglas fir in Germany. We hypothesized that i) trophic niches of generalist predators respond only moderately to native stands and native tree proportion, but respond stronger to non-native Douglas fir presence. We hypothesized further that ii) structural stand parameters such as canopy openness, deadwood availability and herb vegetation complexity shape trophic niches of generalist predators, independent of stand type. Finally, we hypothesized that iii) stand and structural effects on predator trophic niches depend on the location of the site and thus, on environmental conditions like nutrient availability, which fuel lower trophic levels [47, 48].

## Results

### Stand type

#### One-dimensional isotopic metrics

A total of 41 spider species and 22 ground beetle species comprised the top 80% of abundance per plot (Additional file 1: Tables S1, S2). The majority of spider species were web building (n = 29), but size classes were almost evenly balanced for both spiders and ground beetles (Additional file 1: Tables S1, A2).

**Fig. 1:**
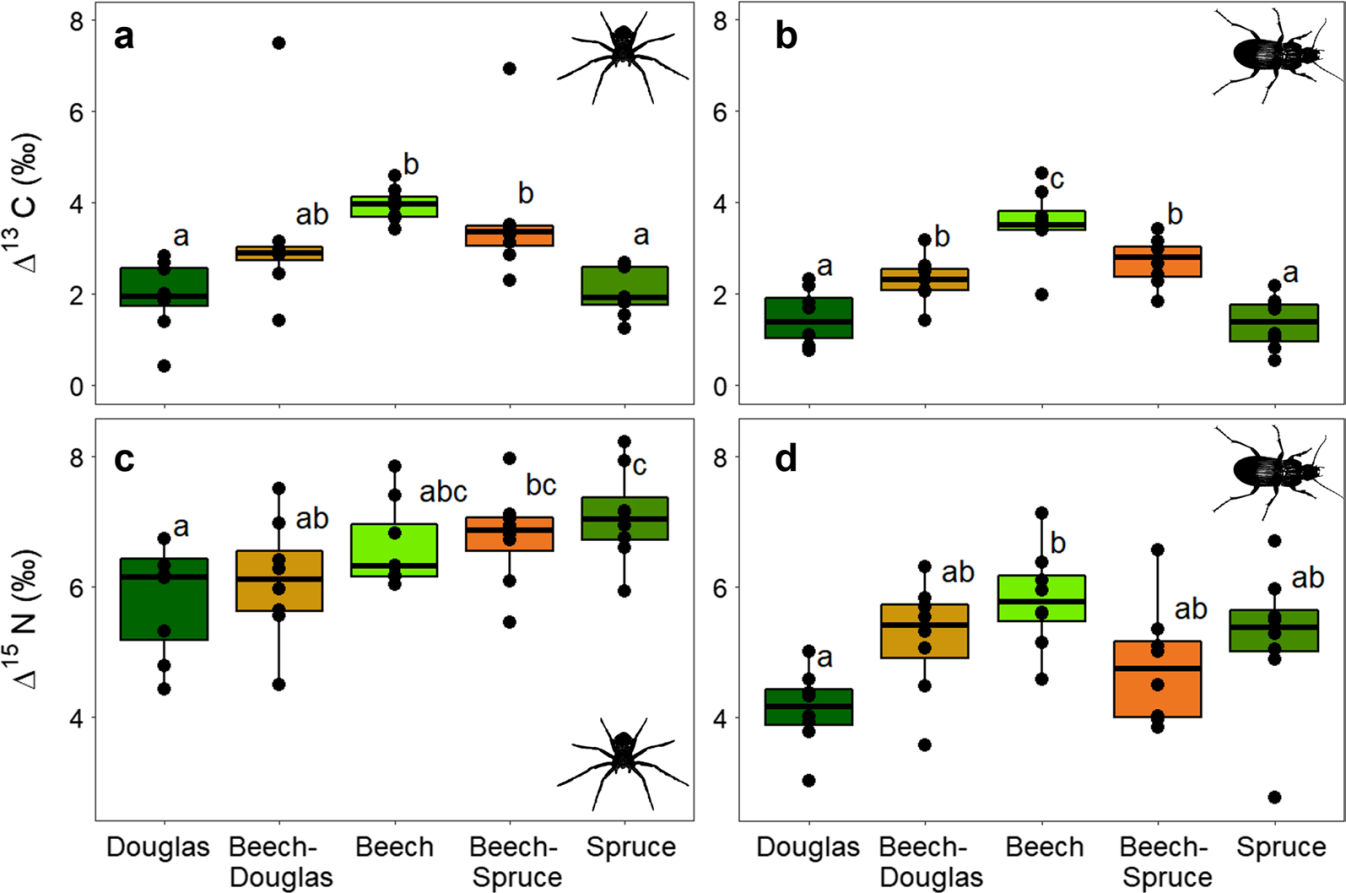
Boxplots of mean ∆^13^C and ∆^15^N values across stand types for **a**, **c** spiders and **b**, **d **ground beetles. Significant differences are marked with lower case letters

For both spiders and ground beetles, *mean* ∆^13^C was significantly higher in pure beech stands than in coniferous stands (F_(4,28)_ = 5.54, p < 0.005 for spiders, F_(4,28)_ = 20.05, p < 0.001 for ground beetles; Fig. 1a, b; Table 1). For spiders, *mean* ∆^13^C in beech was higher by 2.07 ‰ and 2.18 ‰ than in Douglas fir and spruce, respectively. The corresponding differences for ground beetles were 1.99 ‰ and 1.91 ‰. Mixtures represented intermediate values. *Minimum* and *maximum* values of ∆^13^C showed the same pattern. We found no significant differences in ∆^13^C *mean*, *minimum*, *maximum* or *range* between northern and southern sites. Whilst for spiders, the *ranges* of ∆^13^C values did not show any significant differences between stands or north and south, ground beetle *range* values were higher in spruce than in beech monocultures and mixtures, on average by 1.3 ‰ (F_(4,28)_ = 4.52, p < 0.005; Table 1).

**Table 1:** Mean isotopic values of spider (bold) and ground beetle (italics) communities per stand ± standard error

	Douglas	Beech-Douglas	Beech	Beech-Spruce	Spruce	South
Mean ∆^13^C	**1.97 ± 0.28**^**a**^	**3.28 ± 0.63**^**ab**^	**3.96 ± 0.13 **^**b**^	**3.63 ± 0.5**^**b**^	**2.05 ± 0.19**^**a**^	–
	*1.48 ± 0.21*^*a*^	*2.31 ± 0.18*^*b*^	*3.55 ± 0.27*^*c*^	*2.72 ± 0.18*^*b*^	*1.37 ± 0.2*^*a*^	–
Minimum ∆^13^C	**0.89 ± 0.35**^**a**^	**2 ± 0.22**^**b**^	**2.95 ± 0.12**^**b**^	**2.09 ± 0.18**^**b**^	**0.62 ± 0.36**^**a**^	–
	*0.06 ± 0.48*^*a*^	*1.44 ± 0.23*^*b*^	*2.95 ± 0.3*^*c*^	*1.89 ± 0.35*^*b*^	*− 0.15 ± 0.18*^*a*^	–
Maximum ∆^13^C	**3 ± 0.28**^**a**^	**3.88 ± 0.3**^**abc**^	**4.65 ± 0.16**^**c**^	**4.11 ± 0.32**^**bc**^	**3.3 ± 0.25**^**ab**^	–
	*2.44 ± 0.22*^*a*^	*3.08 ± 0.23*^*ab*^	*4.29 ± 0.26*^*c*^	*3.48 ± 0.08*^*bc*^	*2.72 ± 0.27*^*ab*^	–
Range ∆^13^C	**2.11 ± 0.25**^**a**^	**1.88 ± 0.21**^**a**^	**1.7 ± 0.16**^**a**^	**2.02 ± 0.28**^**a**^	**2.68 ± 0.41**^**a**^	–
	*2.37 ± 0.48*^*ab*^	*1.65 ± 0.24*^*a*^	*1.34 ± 0.29*^*a*^	*1.6 ± 0.35*^*a*^	*2.88 ± 0.3*^*b*^	–
Mean ∆^15^N	**5.83 ± 0.31**^**a**^	**6.1 ± 0.32**^**ab**^	**6.63 ± 0.23**^**abc**^	**6.77 ± 0.26**^**bc**^	**7.08 ± 0.25**^**c**^	− 0.9 ± 0.21***
	*4.13 ± 0.21*^*a*^	*5.23 ± 0.3*^*ab*^	*5.81 ± 0.27*^*b*^	*4.8 ± 0.32*^*ab*^	*5.21 ± 0.4*^*ab*^	–
Minimum ∆^15^N	**3.78 ± 0.19**^**a**^	**4.25 ± 0.33**^**a**^	**4.66 ± 0.22**^**a**^	**4.41 ± 0.4**^**a**^	**4.85 ± 0.36**^**a**^	–
	*2.43 ± 0.47*^*a*^	*4.2 ± 0.3*^*bc*^	*4.65 ± 0.43*^*c*^	*3.09 ± 0.49*^*ab*^	*3.02 ± 0.5*^*ab*^	*–*
Maximum ∆^15^N	**7.74 ± 0.51**^**a**^	**8.33 ± 0.53**^**ab**^	**8.63 ± 0.45**^**ab**^	**8.61 ± 0.32**^**ab**^	**9.36 ± 0.47**^**b**^	− 1.59 ± 0.32***
	*5.62 ± 0.33*^*a*^	*6.34 ± 0.97*^*a*^	*7.75 ± 0.92*^*a*^	*6.34 ± 0.29*^*a*^	*6.91 ± 0.42*^*a*^	*–*
Range ∆^15^N	**3.97 ± 0.43**^**a**^	**4.07 ± 0.54**^**a**^	**3.98 ± 0.48**^**a**^	**4.2 ± 0.31**^**a**^	**4.57 ± 0.57**^**a**^	− 1.37 ± 0.37***
	*3.19 ± 0.56*^*a*^	*2.66 ± 0.95*^*a*^	*2.92 ± 1.24*^*a*^	*3.25 ± 0.44*^*a*^	*3.88 ± 0.55*^*a*^	− 1.76 ± 0.8*

Significant differences are indicated in superscript with letters (stand types) or with asterisks (north-south interactions). Significance letters and p-values were received from Tukey HSD post hoc tests of linear mixed-effects models. The column “South” shows significant difference between the southern and northern plots. *P < 0.05; **P < 0.01; ***P < 0.001

*Mean* ∆^15^N showed differing patterns for spiders and ground beetles. Spider ∆^15^N was significantly higher in spruce than in Douglas fir (F_(4,28)_ = 5.28, p < 0.005), on average by 1.25 ‰, whilst mixtures and pure beech stands showed intermediate values (Fig. 1c, Table 1). For ground beetles, spruce comprised 1.08 ‰ higher *mean* ∆^15^N values than Douglas fir but no significance was reached. Yet, in beech stands ∆^15^N values were significantly higher than in Douglas fir (F_(4,28)_ = 4.35, p < 0.01), on average by 1.68 ‰ (Fig. 1d). *Mean* ∆^15^N values of spiders were significantly higher in the northern sites (F_(1,6)_ = 16.91, p < 0.01), on average by 0.9 ‰. *Maximum* ∆^15^N values in the north were higher by 1.59 ‰ (F_(1,6)_ = 24.6, p < 0.01). The *ranges* of ∆^15^N did not differ between stand types for both spiders and ground beetles but differed significantly between north and south in both cases (F_(1,6)_ = 13.95, p < 0.01 for spiders, F_(1,6)_ = 4.8, p < 0.05 for ground beetles). The average *range* of ∆^15^N was higher by 1.37 ‰ and 1.76 ‰ in the north for spiders and ground beetles, respectively (Table 1).

#### Multidimensional isotopic metrics

**Fig. 2:**
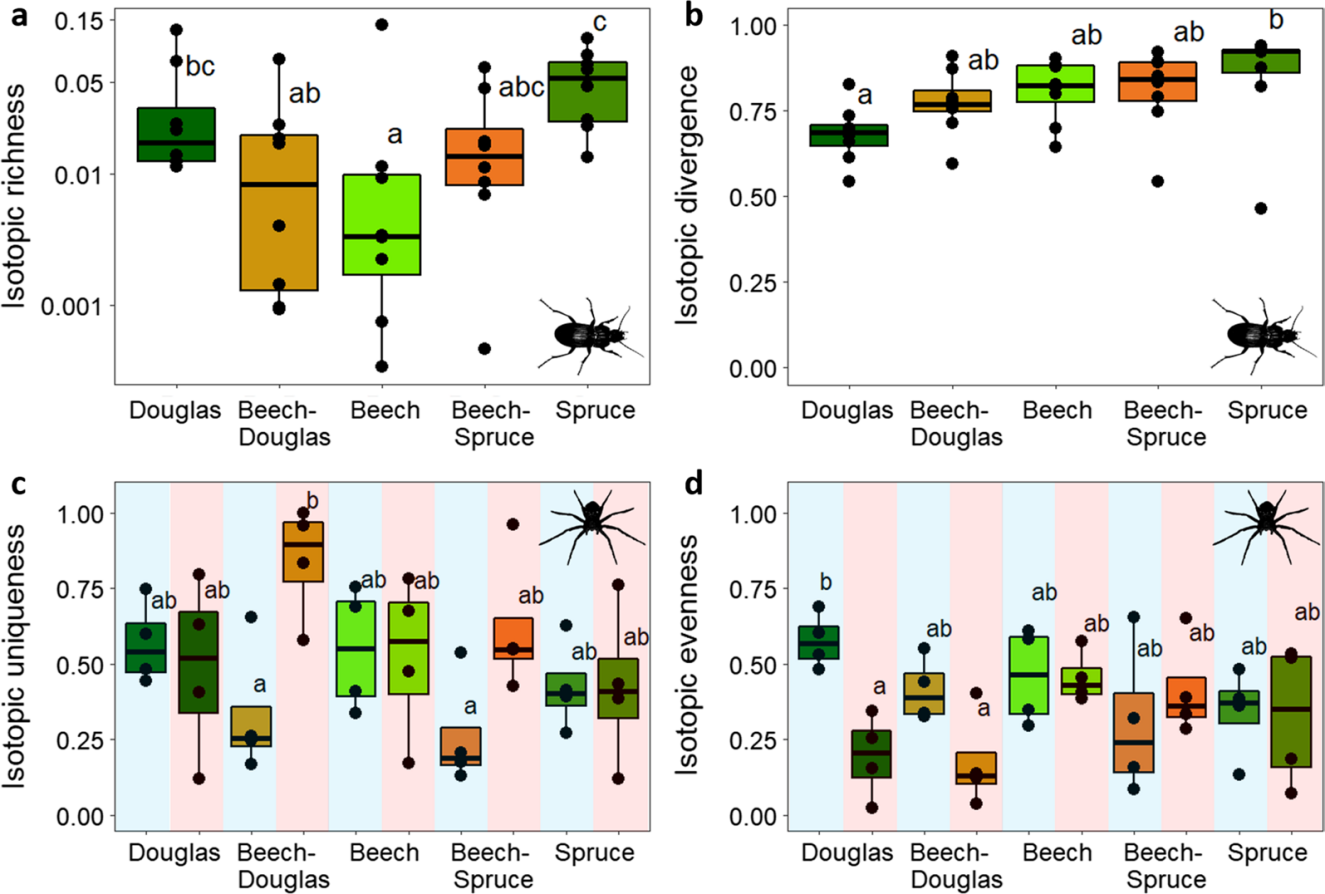
Boxplots of **a** mean isotopic richness (IRic) and **b** isotopic divergence (IDiv) of ground beetles across stand types. **c** Mean isotopic uniqueness (IUni) and **d** isotopic evenness (IEve) of spiders across stand types, divided in northern (light blue) and southern (light red) plots. Significant differences are marked with lower case letters

We found significant differences in multidimensional isotopic metrics of ground beetles between stand types independent of northern or southern location (Table 2). Spruce showed 0.02 higher (scaled value) average isotopic richness than pure beech stands (F_(4,28)_ = 2.43, p < 0.05; Fig. 2a). Further, isotopic divergence was higher by 0.17 (scaled value) in spruce than in Douglas fir (F_(4,28)_ = 2.74, p < 0.05; Fig. 2b).

**Table 2:** Mean multidimensional isotopic metrics of spider (bold) and ground beetle (italic) communities per stand ± standard error

	Douglas	Beech-Douglas	Beech	Beech-Spruce	Spruce	South
IRic	**0.051 ± 0.008**^**a**^	**0.037 ± 0.009**^**a**^	**0.052 ± 0.009**^**a**^	**0.053 ± 0.009**^**a**^	**0.069 ± 0.013**^**a**^	+ 0.153 ± 0.07*
	*0.036 ± 0.014*^*abc*^	*0.017 ± 0.009*^*ab*^	*0.02 ± 0.016*^*b*^	*0.021 ± 0.007*^*bc*^	*0.053 ± 0.011*^*c*^	–
IDiv	**0.655 ± 0.102**^**a**^	**0.707 ± 0.07**^**a**^	**0.751 ± 0.063**^**a**^	**0.643 ± 0.093**^**a**^	**0.614 ± 0.085**^**a**^	–
	*0.682 ± 0.029*^*a*^	*0.772 ± 0.034*^*ab*^	*0.807 ± 0.032*^*ab*^	*0.809 ± 0.043*^*ab*^	*0.851 ± 0.057*^*b*^	–
IDis	**0.258 ± 0.075**^**a**^	**0.257 ± 0.083**^**a**^	**0.411 ± 0.081**^**a**^	**0.232 ± 0.045**^**a**^	**0.297 ± 0.051**^**a**^	–
	*0.549 ± 0.053*^*a*^	*0.562 ± 0.01*^*a*^	*0.537 ± 0.092*^*a*^	*0.606 ± 0.048*^*a*^	*0.634 ± 0.073*^*a*^	–
IEve	**0.385 ± 0.081**^**a**^	**0.295 ± 0.062**^**a**^	**0.456 ± 0.041**^**a**^	**0.359 ± 0.072**^**a**^	**0.334 ± 0.064**^**a**^	–
	*0.664 ± 0.064*^*a*^	*0.604 ± 0.1*^*a*^	*0.645 ± 0.072*^*a*^	*0.632 ± 0.038*^*a*^	0.602 ± 0.06^*a*^	–
IUni	**0.528 ± 0.076**^**a**^	**0.587 ± 0.117**^**a**^	**0.536 ± 0.078**^**a**^	**0.441 ± 0.096**^**a**^	**0.425 ± 0.067**^**a**^	+ 0.153 ± 0.077*
	*0.564 ± 0.071*^*a*^	*0.556 ± 0.09*^*a*^	*0.626 ± 0.07*^*a*^	*0.711 ± 0.06*^*a*^	*0.689 ± 0.048*^*a*^	–

*IRic* isotopic richness, *IDiv* isotopic divergence, *IDis* isotopic dispersion, *IEve* isotopic evenness, *IUni* isotopic uniqueness

Significant differences are indicated in superscript with letters (stand types) or with asterisks (north-south interactions). Significance letters and p-values were received from Tukey HSD post hoc tests of linear mixed-effects models. The column “South” shows significant difference between the southern and northern plots. *P < 0.05; **P < 0.01; ***P < 0.001

Comparing north and south revealed more differences: isotopic richness tended to be higher in the north, with the greatest difference (F_(9,23)_ = 3.7, p < 0.01) in ground beetles between beech in the south (0.004; scaled value) and spruce in the north (0.06). For spiders, isotopic divergence and isotopic uniqueness in the south were significantly higher by 0.17 and 0.15 respectively, than in the north (scaled values; F_(1,6)_ = 6.38, p < 0.05 for isotopic divergence, F_(1,6)_ = 3.99, p < 0.05 for isotopic uniqueness). Isotopic uniqueness of spiders was lowest in both beech-conifer mixtures in the north, whereas in beech-Douglas fir mixtures in the south it was higher by 0.54 (scaled value; F_(9,23)_ = 4.22, p < 0.01; Fig. 2c). Isotopic evenness of spiders differed significantly between Douglas fir monocultures in the north and south (F_(9,23)_ = 4.22, p < 0.01) with values higher by 0.38 in the north (scaled value; Fig. 2d).

### Environmental variables

#### One-dimensional isotopic metrics

**Fig. 3:**
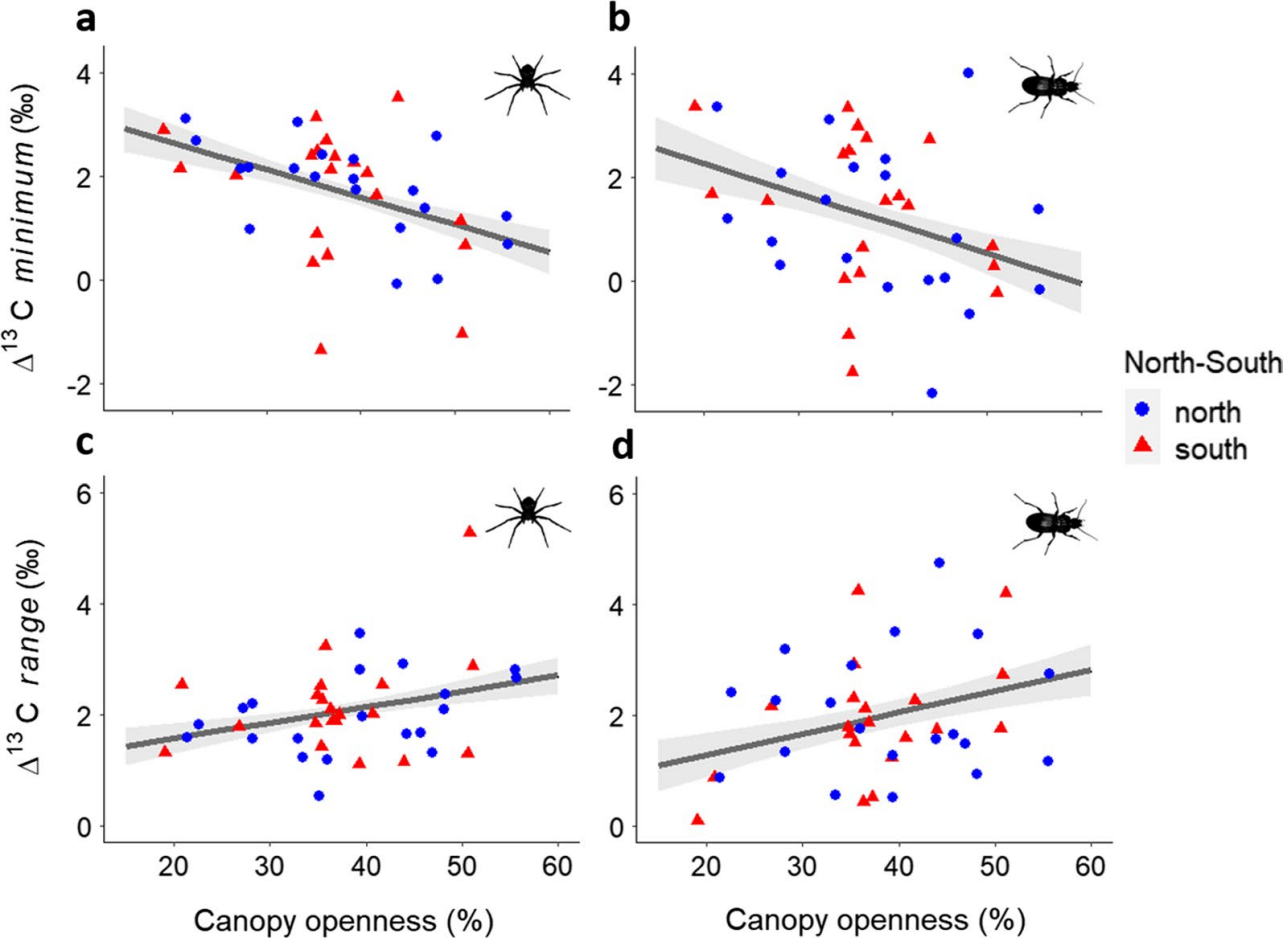
Relationships between *minimum* and *range* ∆^13^C values and canopy openness for **a**, **c** spiders and **b, d** ground beetles. Grey bands represent 95% confidence intervals. Values from the northern plots in blue dots and southern plots in red triangles

**Fig. 4:**
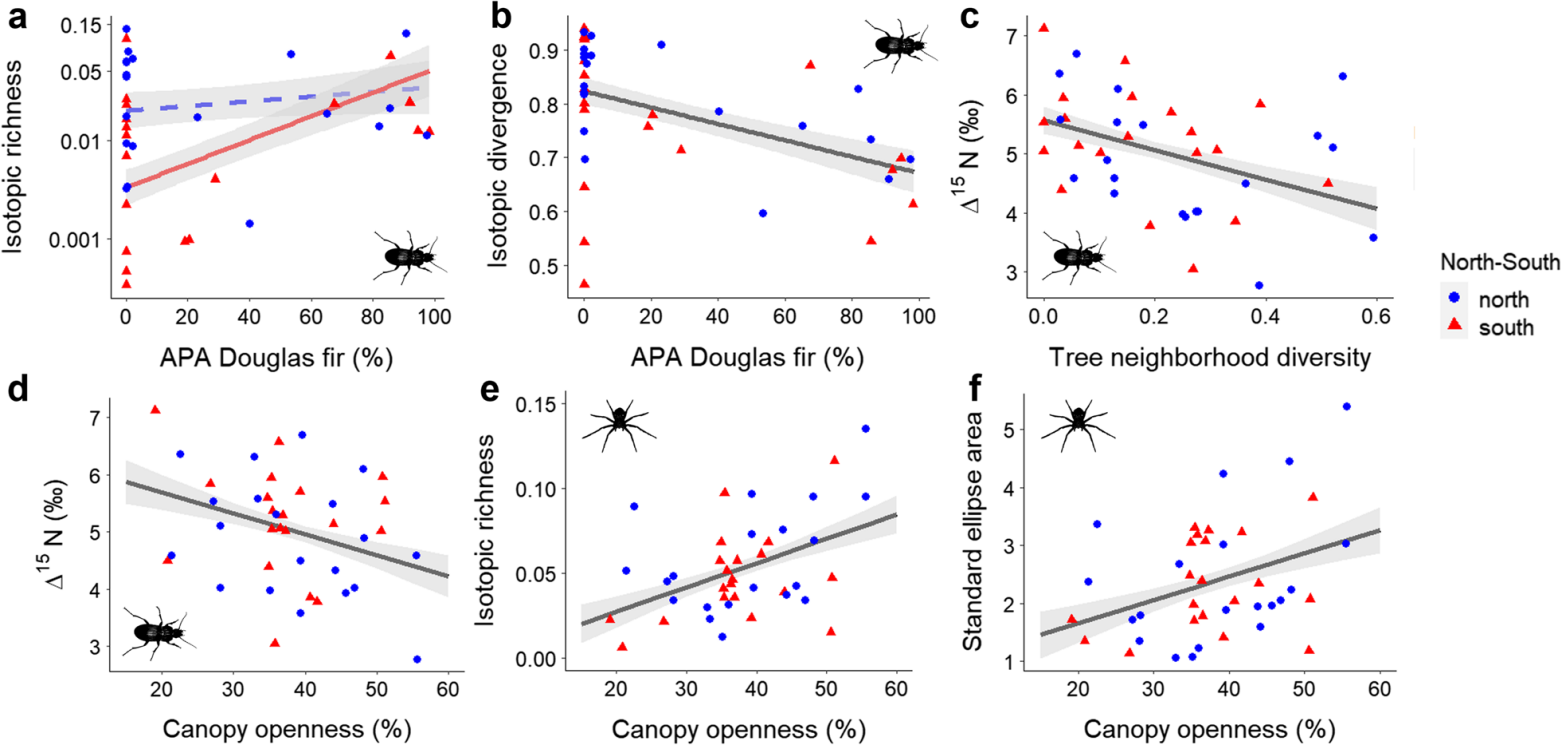
Relationships between Douglas fir proportion (APA) and **a** isotopic richness (IRic) and **b** isotopic divergence (IDiv) of ground beetles; **c** tree neighborhood diversity (NDiv) and ground beetle *mean* ∆^15^N; canopy openness and **d** ground beetle *mean* ∆^15^N values, **e** isotopic richness (IRic) of spiders and **f** standard ellipse area (SEA_c_) of spiders. Grey bands represent 95% confidence intervals. Significant relationships with solid lines, non-significant regressions dashed. Values and regression line from the northern plots in blue and southern plots in red

*Mean*, *minimum* and *maximum* ∆^13^C values of spider and ground beetle communities correlated negatively with increasing APA proportions of both conifers (Additional file 1: Tables S3–S6). This effect was independent of northern and southern location, except for the ∆^13^C maxima of spiders, which did not correlate with Douglas fir APA in the north. Further, canopy openness correlated negatively with *minimum* ∆^13^C values of both predator communities (Fig. 3a, b), and herb vegetation complexity decreased *mean* ∆^13^C values of ground beetles in the north (Additional file 1: Table S6). *Ranges* of ∆^13^C showed contrasting patterns to *mean* ∆^13^C values. Canopy openness and conifer APAs correlated positively with the community ∆^13^C *ranges* (Fig. 3c, d; Additional file 1: Tables S3–S6)*. Mean* and *minimum* ∆^15^N values were correlated negatively with Douglas fir APA (Additional file 1: Tables S3, S5). Ground beetle *mean* and *minimum* ∆^15^N values further correlated negatively with canopy openness and tree neighborhood diversity (Ndiv; Fig. 4c, d). Moreover, spruce APA lowered *minimum* ground beetle ∆^15^N values and herb vegetation complexity lowered *minimum* spider ∆^15^N values. Ground beetle ∆^15^N *ranges* increased with increasing spruce APA (Additional file 1: Tables S3–S6).

#### Multidimensional isotopic metrics

Especially in the southern sites, isotopic richness (IRic) correlated positively with spruce and Douglas fir APA for ground beetles (Fig. 4a, Additional file 1: Tables A3-A6). Spider IRic was positively correlated with canopy openness (Fig. 4e). Isotopic divergence (IDiv) of ground beetles correlated negatively with Douglas fir APA (Fig. 4b). Isotopic dispersion of spiders was correlated negatively with total deadwood volume in the southern sites (Additional file 1: Table S4). Also exclusively in the south, but positively correlated was spider isotopic uniqueness (IUni) with ground vegetation complexity (Additional file 1: Table S4). Standard ellipse area (SEA_c_) analyses were only carried out for spiders due to the small number of species comprising the top 80% of ground beetle abundances. Spider SEA_c_ was positively correlated with canopy openness, regardless of northern or southern location (Fig. 4f).

### Size classes and hunting mode

**Fig. 5:**
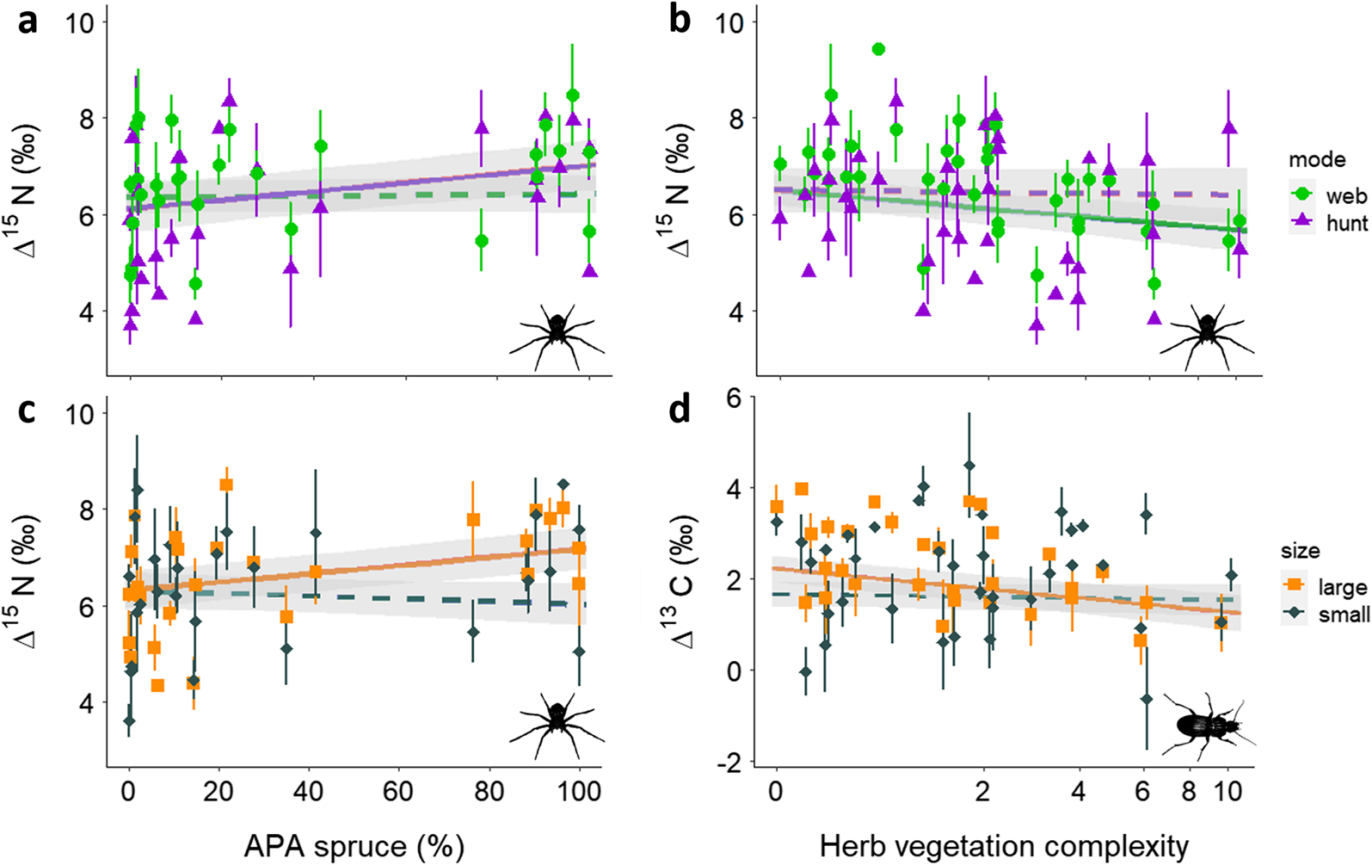
Relationships between *mean* ∆^15^N values of spiders and **a** proportion of Norway spruce (APA) and **b** herb vegetation complexity in consideration of spider hunting mode. **c** Relationship between *mean* ∆^15^N values of spiders and proportion of Norway spruce (APA) under consideration of spider size class. **d** Relationship between *mean* ∆^13^C values of ground beetles and herb vegetation complexity under consideration of ground beetle size class. Values of web hunters in green dots; hunting spiders in violet triangles; large species in orange squares and small species in grey diamonds. Grey bands represent 95% confidence intervals in all plots

Size class and hunting mode of the studied organisms showed no significant differences in ∆^13^C and ∆^15^N depending on stand type. However, ∆^15^N values of large spiders and ground hunting spiders were correlated positively with Norway spruce APA (Df = 47.43, t = 2.7, p < 0.01 for large spiders, Df = 77.16, t = 2.5, p < 0.05 for ground hunting spiders; Fig. 5a, c). By contrast, ∆^15^N values of web building spiders were lowered by increasing herb vegetation complexity (Df = 46.79, t = − 2.33, p < 0.05; Fig. 5b). Large ground beetle and web building spider ∆^13^C correlated negatively with herb vegetation complexity (Df = 68.42, t = − 2.73, p < 0.01 for large beetles, Df = 39.67, t = − 1.97, p = 0.051 for web building spiders; Fig. 5d).

## Discussion

Our study shows that adding conifers, particularly non-native Douglas fir, to European beech stands affects the trophic niche structure of forest floor-associated arthropod predators. Higher beech proportions promoted ∆^13^C, indicating a more decomposer-based resource use. Conifer species presence as well as increasing canopy openness and herb complexity increased isotopic *ranges* and isotopic richness. Moreover, there were significant differences between Douglas fir and native tree species, with shorter food chains in Douglas fir (∆^15^N). The generalist predator community in Douglas fir also was less specialized (isotopic divergence) than communities in native forest stands, especially under beneficial abiotic conditions in southern sites (isotopic evenness). As beech-conifer tree mixtures mitigated these effects, we emphasize the need to foster mixed stands of broadleaves and conifers in forest management strategies, whilst adding only small proportions of non-native tree species.

### One-dimensional isotopic metrics

In our study, ∆^13^C and ∆^15^N of aboveground arthropod predators were more strongly influenced by stand type than past studies suggested. Whereas ∆^13^C differed mostly between European beech and coniferous stands, ∆^15^N was lowest in non-native Douglas fir. The pattern of increasing *mean* ∆^13^C relative to the litter source (“detrital shift”; [49]) from conifers over mixtures to beech is widespread for coniferous and deciduous trees [41, 42, 47]. Lower detrital shift in coniferous than in deciduous forests is explained by the almost impenetrably thick leaf litter layers in coniferous stands. This impenetrability limits the connection between above-ground arthropods and the root-fueled microbial food webs with high ∆^13^C signatures [41, 42]. Consequently, low ∆^13^C values are connected to a more plant- and herbivore-fueled food web [28, 50]. This assumption is supported by our finding that increasing canopy openness, and the correspondingly greater herb complexity, correlated positively with both conifer proportions and low ∆^13^C. In line with these findings, Matevski et al. [32] found higher arthropod attack rates on herbivores on the same study plots in coniferous stands than in beech. European beech stands, in turn, were found to have more microbial and macro-decomposer activity than coniferous stands [44, 51], which increases the input of high ∆^13^C into the food web.

The ∆^13^C *ranges* of spiders in our study remained stable across stand type categories, supporting findings of relatively stable use of resources across stand types [39, 43, 47]. However, ∆^13^C *ranges* were wider with increasing conifer proportions and canopy openness within the stand type categories, especially when considering ground beetles and spruce. Wide *ranges* of ∆^13^C can display a broader range of available resources, but they might also reflect a high diversity of arthropod predators themselves, as found in coniferous stands compared to European beech [19, 24]. The second possibility, however, can only be inferred if the predator diversity corresponds to functional diversity in terms of prey use—but ground beetles are mostly generalists, and species richness of ground beetles does not necessarily reflect their functional impact [52]. It is therefore more likely that coniferous stands with open canopies provide a wider range of resources for generalist predators than beech stands.

We found lower ∆^15^N *means* in non-native Douglas fir stands than in native spruce (spiders) or native beech stands (ground beetles). This result highlights Douglas fir having different food webs than native tree species, which could be explained by the fact that arthropod communities in Douglas fir differ from those in European beech and Norway spruce [22, 23]. It is particularly interesting that *mean* ∆^15^N was low in the Douglas fir stands of our study. This is usually not the case for conifers, as low ∆^15^N is typical for broadleaved stands compared to low values in coniferous stands [53]. Low ∆^15^N values are commonly explained by increased availability of low trophic levels like microbes and decomposers in deciduous forest sites, which then fuel the higher trophic levels [40, 41], whilst, again, thick leaf litter layers in coniferous stands restrict the access to low trophic levels [41, 42]. However, as ∆^13^C values in Douglas fir were lower than in beech and did not differ from spruce, it seems unlikely that Douglas fir provides enhanced access to the microbial food web. Our results rather suggest that food chains are shorter in Douglas fir [54]. In this context, particularly high ∆^15^N averages of spiders in Norway spruce imply increased intra-guild predation, as spruce does not provide as much herb cover and therefore potential herbivore prey as Douglas fir [19], whilst also not providing access to microbial food webs as beech [41]. These findings highlight from a functional perspective that arthropod communities in non-native Douglas fir differ significantly from those in native Norway spruce [21, 22, 55]. Therefore, despite the current diebacks of Norway spruce [5], replacement of native conifers with non-native species should be limited, to avoid extensive alteration and simplification of predator food web structure in forests.

*Mean* and *minimum* ∆^15^N of ground beetles correlated negatively with tree neighborhood diversity, which indicates increased access to lower trophic levels and thus, less intraguild-predation pressure for generalist predators when increasing stand diversity [24]. Notably, *mean* and *maximum* ∆^15^N were highest for spiders in the northern sites. Also, ∆^15^N *ranges* were wider in the north than in the south for both spiders and ground beetles. Large *ranges* of ∆^15^N reflect multiple trophic levels [28, 56] and, similarly to high *mean* ∆^15^N values, they can be either assigned to intra-guild predation or more trophic levels in the available resources [56]. As the dry conditions in the northern sites increase susceptibility for stress in the microbial food web [44], we suggest that the challenging conditions increase intraguild predation [57].

### Multidimensional isotopic metrics

Isotopic richness and weighted multidimensional isotopic metrics of spiders and ground beetles mostly remained stable across stand types and environmental conditions, indicating a relatively stable trophic niche. Stable trophic niches across similar ecosystems were also observed in soil arthropods [49]. However, we found some fine-scale interactions which provided deeper insight into modulations of trophic niche structure by forest stand types and environmental conditions.

Arthropod isotopic richness (IRic) generally responded positively to the presence of spruce, whereas Douglas fir had intermediate values and beech had the lowest values. Higher diversity of generalist arthropod predators in coniferous stands compared to beech has previously been shown for our and other study sites [19, 24]. Especially rare species might increase IRic in coniferous stands [19]. Rare species with functionally deviating trophic niches increase the convex hull because IRic is not weighted by relative biomass [45]. The positive effect of spruce on IRic was especially pronounced for ground beetles, whereas spider IRic responded positively to canopy openness. Similarly, spider stable isotope values covered larger standard ellipse areas with increasing canopy openness. This is in line with earlier studies, stating strong interrelations of spider communities and canopy openness and herb structure [58–60]. Canopy openness increases herb vegetation availability and complexity and thus, provides necessary structures for web-weavers and hideouts for hunting spiders [59–61].

Isotopic divergence (IDiv) of ground beetles was significantly higher in spruce than Douglas fir stands and Douglas fir proportion had a negative effect on IDiv. Low IDiv reflects few isotopic values that deviate from the average isotopic values across the entire community and therefore supports findings of few specialized arthropod species in stands of non-native trees [22, 62].

Available dead wood lowered spider isotopic dispersion (IDis) in the southern sites. Indeed, total deadwood volume was highest in the southern sites. We interpret this as a carbon source, which, if increasing, promotes dominance of one feeding type [28, 45]. This highlights that not only ground beetles, but also spider assemblages are affected by deadwood availability [63].

Whereas spider communities in the northern, sandy Douglas fir sites comprised high isotopic evenness (IEve) values and thus different trophic positions were evenly distributed, there seemed to be a dominant feeding mode in the loamy southern sites with higher precipitation, where IEve was significantly lower. Since the ∆^15^N *range* was generally higher in the north, we hypothesize that scarce resources in the north promote niche differentiation and exploitation of various food sources [64, 65], possibly including intra-guild predation—and that this effect is especially pronounced for non-native Douglas fir. This supports findings of distinct (negative) responses of Douglas fir-associated arthropod communities under stressful abiotic conditions, compared to native tree species [44]. Under beneficial conditions, however, Douglas fir seems to host uniform and unspecialized communities, supporting the findings of low isotopic divergence in Douglas fir and previous studies showing low degrees of arthropod specialization in non-native trees [22, 62]. These findings, again, highlight that extensive replacement of native conifers with non-native species should not be recommended, as it simplifies trophic community structures of arthropod predators.

Low IUni values of spiders in the northern sandy plots can either be interpreted as low complexity of trophic organization and thus lower stability [65], or as high trophic redundancy and thus, enhanced ecosystem stability [28]. Our results and a previous study [44] rather apply to Hutchinson’s interpretation [65], as the sandy and dryer northern sites were the more challenging environment for arthropod communities.

### Size classes and hunting mode

Tree species proportion and structural stand parameters had different impacts on generalist predators depending on size and hunting strategy, highlighting that even for generalists, life-history strategies can modify relationships between forest management and trophic niche structure. Herb vegetation complexity had a stronger impact on large ground beetles, lowering the ∆^13^C values with increasing complexity significantly stronger than those of small ground beetles. This indicates that large ground beetles are more linked to herbivore prey if vegetation complexity increases, whereas small ground beetles respond neither to increased decomposer nor to herbivore availability. In accordance to these findings of minor importance of decomposer prey to ground beetles, ground beetle density is not linked to increased decomposer prey availability [66].

Web-building spiders showed negative ∆^13^C and ∆^15^N responses to increasing herb complexity, indicating both a switch from decomposer prey to herbivores and decreased intra-guild predation if ground vegetation is present. This reflects the dependence on available structure for web-weavers [58, 61]. Ground hunter and large spider ∆^15^N responded positively to increasing spruce proportion. Ground hunters are highly abundant in spruce forests and the limited access to decomposer prey promotes inclusion of intra-guild prey into their diet [67].

## Conclusions

Trophic niches of aboveground generalist predators were strongly influenced by forest stand type and tree species identity. While effects of Douglas fir on trophic niche structure of arthropod predators show many similarities to effects of native conifers (less decomposer-fueled than beech; canopy openness promotes isotopic richness), closer inspection uncovers simplifying and possibly adverse effects of non-native Douglas fir on some niche properties (shorter food chains; low isotopic divergence and evenness). Tree mixtures generally mitigated differences of trophic niches between broadleaved and coniferous stands. This emphasizes that tree mixtures do not necessarily have beneficial effects over average monoculture effects (as often found for functions such as productivity; e.g. [6]), but can rather cause averaging effects [68]. Nevertheless, this averaging can be used to balance economic interests and conservation of functional diversity. We therefore recommend only small-scale replacement of native conifers with their non-native counterparts, preferably in mixed stands with native broadleaves like European beech. Additionally, forest management might promote top-down control via increasing canopy gaps.

## Methods

### Sampling sites

The study was conducted in the federal state of Lower Saxony in northwest Germany. The 40 study plots were located in eight study sites, with each site comprising five different forest stands (plot “quintets”; [69]). These stands were pure stands of native European Beech (*F. sylvatica*), native Norway Spruce (*P. abies*), and non-native Douglas fir (*P. menziesii*), and mixed stands of beech-Douglas fir and beech-spruce. Within sites, distances between plots ranged from 76 to 4600 m. Each of the 40 plots had a size of 0.25 ha. The eight sites were divided into four northern sites and four southern sites [69]. Between site distances ranged from 5 to 190 km, with 105 km as the minimum distance between northern and southern sites. This division of sites allows testing for effects of different environmental conditions of forest stands, as site characteristics vary between the two regions. In the northern, sandy sites, precipitation is lower (708 mm mean annual precipitation) and the soil is nutrient-poor due to dry dystrophic sand deposits [70]. The southern, loamy sites have higher precipitation (888 mm mean annual precipitation) and are richer in nutrients due to their spodic cystric cambisols soil characteristics [70]. Tree ages were 80 years on average, ranging from 43 to 131 years [32].

### Arthropod data

Between beginning of April and mid-June 2019, we placed twelve pitfall traps (upper diameter 9.4 cm, volume 0.5 L) in a 3 × 4 grid and with 10 m distance between traps on each plot. We covered the traps with a metal mesh (mesh size 1.5 cm) to reduce vertebrate bycatch. The trapping solution was 150 ml of a 50:50 mixture of propylene glycol and water. We collected the traps every three weeks, resulting in three sampling periods and a total of 61 trapping days. Further details of the sampling method are given in [19]. Samples were stored in 80% ethanol. For further analysis, we reduced the sampling size to eight of the twelve pitfall traps per plot (except for ground beetles in the southern sites, which were identified in all twelve traps for a previous study, see [19]). As we used relative abundance and relative biomass in our analyses, the reduced set of eight traps can be considered as equally representative as the full set of twelve traps. Spiders were identified to species level following Nentwig et al. [71]. Ground beetles were identified to species level following Müller-Motzfeld [72]. We retrieved mean body length values of spider species from Nentwig et al. [71] and calculated the mean dry body mass of each species, using the linear regression model as established by Penell et al. [73]. For ground beetles, we used mean body length values from Müller-Motzfeld [72] and calculated the mean fresh biomass with the formula ln *y* = − 8.92804283 + 2.5554921 × ln *x,* with y being the body mass and x the body length [74]. We then calculated the relative biomass per plot of all spider and ground beetle species, respectively. We divided both spiders and ground beetles into two size classes, with spider species < 1 mg mean individual dry biomass as “small” and species ≥ 1 mg as “large”. The threshold for ground beetles was 100 mg mean individual fresh biomass. We retrieved the spider hunting strategies from Nentwig et al. [71] and categorized them as either ground hunters or web builders.

### Habitat structure

We used several structural parameters to relate trophic niche metrics to stand characteristics on plot level: relative area potentially available (APA) of Douglas fir and Norway spruce as a measure of tree proportions, neighborhood diversity (NDiv), canopy openness, herb vegetation complexity and deadwood volume. We included relative tree proportions to account for different mixture proportions in our plots. For the calculation of APA, plots were divided into pixels, which then were assigned to the closest trees, whilst the trees are weighted with their size [75]. NDiv accounts for possible effects of tree diversity. NDiv is a novel index of spatially explicit diversity, using the number of monospecific and heterospecific neighbors bordering the APA of each tree. This individual tree-based calculation avoids high scores for plots with monospecific patches of different tree species [76]. NDiv values range between 0 (monospecific) and 1 (heterospecific). Canopy openness, herb vegetation complexity and deadwood volume are important community-shaping parameters for forest floor-associated arthropods [19, 60, 63]. We measured canopy openness at the center of each trap using a Solariscope (SOL 300, Ing.-Büro Behling, Hermannsburg). For herb vegetation structure we divided each 10 × 10 grid cell around the traps into 4 quartiles and measured each quartile. We assessed herb vegetation complexity by counting all points where plant material touched or intercepted strings of 30 cm length at the heights of 3, 10, 20, 30, 40 and 50 cm. For total deadwood volume (m^3^/ha), we measured all stumps and logs with a diameter > 5 cm across the entire sampling grid. All structural parameters were averaged on the plot level.

### Stable isotope analysis

We analyzed all species comprising the top 80% of spider or ground beetle abundance in each plot. This ensured the depiction of the “core” community and thus its effective functional role [28]. In case of multiple species with the same abundance adding up to the top 80%, we chose the species with higher mean body mass according to [71]. Prior to stable isotope analysis, we separated the prosoma of spiders and the legs of ground beetles and dried them at 60 °C for 72 h. We chose these body parts to ensure sufficient minimum weight and to exclude isotopic bias by recently digested material [77, 78]. In case of large species, we used only half of the prosoma (spiders) or half the femur (ground beetles). For very small species, we pooled either multiple prosomas or legs to reach the required minimum weight of ~ 0.05 mg.

Isotopic analysis of carbon and nitrogen was performed by the Centre for Stable Isotope Research Analysis at the University of Göttingen, using an elemental analyzer (CE-Instruments, Rodano, Milano, Italy) coupled with an isotope ratio mass spectrometer (Delta XP, Thermo Electron, Bremen, Germany). Small samples < 0.1 mg were analyzed with an adapted setup [79]. Isotope values were denoted as deviation δ relative to a standard [80], with δ^15^N and δ^13^C being defined as δX (‰) = (R_sample_ – R_standard_)/R_standard_ × 1000. R represents the respective ratio between heavy and light isotope (^13^C/^12^C or ^15^N/^14^N). The used standards were Vienna PeeDee belemnite (C) and atmospheric nitrogen (N). Internal calibration was done with acetanilide (C_8_H_9_NO, Merck, Darmstadt). Arthropod isotopic ratios δ^13^C and δ^15^N were calibrated with mean δ values of leaf litter from the respective plot to compensate for inter-site variation in the isotopic baseline [47]. For baseline calibration, litter δ^13^C and δ^15^N values were subtracted from the respective arthropod isotopic ratios. The calibrated ratios are denoted as Δ^13^C and Δ^15^N. Seven of the 40 study plots were replaced after litter sampling in 2018 due to storm damage. Therefore, the baseline of seven plots was collected in proximate stands of the same type.

We calculated all isotopic metrics on the level of community per plot for spiders and ground beetles separately. The calculated one-dimensional isotopic metrics were: isotopic *mean*, *minimum*, *maximum* and *range* of Δ^13^C and Δ^15^N. For the multidimensional isotopic metrics, we first calculated the unweighted standard ellipse area (SEA_c_), using the “SIBER” R package [46]. Using the code of Cucherousset and Villéger [45], we then calculated the unweighted isotopic richness (IRic) and the relative biomass-weighted isotopic divergence (IDiv), isotopic dispersion (IDis), isotopic evenness (IEve) and isotopic uniqueness (IUni). SEA_c_ is a smoothed significance ellipse, derived from the hull of all ∆^13^C and ∆^15^N values, displaying the trophic niche of the study organism [37, 46]. IRic estimates the total level of trophic diversity of communities based on the isotopic niche space occupied. Values between 0 and 1 indicate the space filled by the studied species across the community. IDiv measures the distance between all studied species within the convex hull area. Values close to 0 indicate that extreme values are rare, whilst values close to 1 represent dominance of species with extreme values (e.g. species with a specialized diet). IDis combines IDiv and the convex hull area, resulting in a multidimensional variance. If species of contrasting stable isotope values dominate, IDis approaches 1 (e.g. top predators versus primary producers or predation on herbivores versus predation on decomposers), whereas it approaches 0 when most species are positioned near the community’s center of gravity. IEve quantifies the distribution in the stable isotope space and accounts for distances between values. Evenly distributed community values are represented by IEve values close to 1, whilst clustered isotopic values push IEve towards 0 (e.g. if most species feed on the same source). Finally, IUni evaluates whether isotopic values tend to be unique or if they overlap. IUni values close to 1 indicate uniqueness of most isotopic values and IUni values close to 0 indicate high redundancy of isotopic values (e.g. if one trophic position is covered by multiple species and the loss of one species would not change the system significantly). For further technical details of multidimensional isotopic metrics see [45].

### Data analysis

All statistical analyses were conducted in R 4.1.0 [81]. We analyzed the trophic community structures of spiders and ground beetles by using both one-dimensional isotopic metrics of Δ^13^C and Δ^15^N (*mean*, *minimum*, *maximum*, *range*) and multidimensional isotopic metrics [37, 45, 46].

We divided the analysis into two different linear mixed-effects model types. First, we compared forest stand types as categorical predictor variable with the community isotopic metrics as the response variables (Tables 1, 2). For the second model approach, we replaced the categorical predictor variable with the numerical tree species proportions and habitat structure variables. Additionally, we included the interaction between predictor variables and the two geographic study regions (north and south) as fixed effects in both model types. For analyses of hunting mode and size class, we did not use community-level but species-level isotopic values. We conducted all analyses at plot-level, with the eight geographically distinct sites included as random effect. In case of species level isotopic metrics, we included all plots nested in sites and species as crossed random effects.

Linear mixed-effects models at community level were run using the R package “nlme” [82] and, for models on hunting type using crossed random effects, with the package “lme4” [83]. We checked all models for normal and homoscedastic residual distribution and if needed, responses were log-transformed. We used the variance inflation factor (VIF) to check for multicollinearity between co-variables [84]. In case of VIFs > 5, we adjusted the predictor choice. We split the model with numerical predictors into one tree proportion model and one habitat structure model to avoid collinearity between conifer proportions and the structural parameters openness and herb vegetation complexity. After final predictor choice, we ran a stepwise selection approach based on the Akaike Information Criterion (AIC), using the “MASS” R package [85]. This selection reduces the models to the smallest global AIC and thus, the essential predictors [86]. For models with the categorical response of stand type, we used a Tukey HSD post hoc test to inspect significant differences, using the “multcomp” R-package [87]. All results were visualized using the “ggplot2” R-package [88]. In case of co-variable dependent skewness of the response, we log-transformed the respective co-variable and reran the model to avoid disproportionally high weighting for extreme values [89].

## Supplementary Information

**Additional file 1: Table S1**: Spider species contributing to the analyzed top 80 % of most abundant spider species per plot. **Table S2**: Ground beetle species contributing to the analyzed top 80 % of most abundant ground beetle species per plot. **Table S3**: Model summaries for the spider community metrics versus proportions of conifers (APA Douglas fir & APA Norway spruce) with north-south interaction. **Table S4**: Model summaries for the spider community metrics versus herb vegetation complexity (Herb complexity), canopy openness, total deadwood volume and neighborhood diversity (NDiv) with north-south interaction. **Table S5**: Model summaries for the ground beetle community metrics versus proportions of conifers (APA Douglas fir & APA Norway spruce) with north-south interaction. **Table S6**: Model summaries for the ground beetle community metrics versus herb vegetation complexity (Herb complexity), canopy openness, total deadwood volume and neighborhood diversity (NDiv) with north-south interaction.

## Data Availability

Data will be made available via https://www.pangaea.de/ after acceptance.
