# Correction to: Conifers and non-native tree species shift trophic niches of generalist arthropod predators in central european beech forests

**DOI:** 10.1186/s12862-023-02109-x

**Published:** 2023-03-27

**Authors:** Benjamin Wildermuth, Riko Fardiansah, Dragan Matevski, Jing‑Zhong Lu, Peter Kriegel, Stefan Scheu, Andreas Schuldt

**Affiliations:** 1 Forest Nature Conservation, University of Gottingen, Busgenweg 3, 37077 Gottingen, Germany; 2 Johann‑Friedrich‑Blumenbach Institute of Zoology and Anthropology, University of Gottingen, Untere Karspule 2, 37077 Gottingen, Germany; 3 Department of Animal Ecology and Tropical Biology (Zoology III), Field Station Fabrikschleichach, University of Wurzburg, Glashuttenstrase 5, 96181 Rauhenebrach, Germany; 4 Centre of Biodiversity and Sustainable Land Use, University of Gottingen, Busgenweg 1, 37077 Gottingen, Germany

**Correction to:** ***BMC Ecol Evo*** **23, 3 (2023)**

10.1186/s12862-023-02105-1

Following publication of the original article [1], the authors identified an error in the authors’ names. The given name and family name were erroneously transposed.

The incorrect authors’ names are: Wildermuth Benjamin, Fardiansah Riko, Matevski Dragan, Lu Jing‑Zhong, Kriegel Peter, Scheu Stefan and Schuldt Andreas.

The correct authors’ names are: Benjamin Wildermuth^1*^, Riko Fardiansah^1^, Dragan Matevski^1^, Jing‑Zhong Lu^2^, Peter Kriegel^1,3^, Stefan Scheu^2,4^ and Andreas Schuldt^1,4*^.

The author group has been updated above and the original article [1] has been corrected.
