# Supplementary material for: Conifers and non-native tree species shift trophic niches of generalist arthropod predators in Central European beech forests
Source: BMC Ecol Evol. 2023 Feb 3;23:3. doi: 10.1186/s12862-023-02105-1 (PMC9896740; doi:10.1186/s12862-023-02105-1)
Supplement: Supplementary file 1 — Additional file 1: Table S1: Spider species contributing to the analyzed top 80 % of most abundant spider species per plot. Table S2: Ground beetle species contributing to the analyzed top 80 % of most abundant ground beetle species per plot. Table S3: Model summaries for the spider community metrics versus proportions of conifers (APA Douglas fir & APA Norway spruce) with north-south interaction. Table S4: Model summaries for the spider community metrics versus herb vegetation complexity (Herb complexity), canopy openness, total deadwood volume and neighborhood diversity (NDiv) with north-south interaction. Table S5: Model summaries for the ground beetle community metrics versus proportions of conifers (APA Douglas fir & APA Norway spruce) with north-south interaction. Table S6: Model summaries for the ground beetle community metrics versus herb vegetation complexity (Herb complexity), canopy openness, total deadwood volume and neighborhood diversity (NDiv) with north-south interaction. [file 12862_2023_2105_MOESM1_ESM.docx]

# **Additional file 1**

Additional File 1: Table S1 Spider species contributing to the analyzed top 80 % of most abundant spider species per plot. Species < 1 mg mean individual dry biomass were categorized as s = small and species ≥ 1 mg were categorized as l = large. In the “plots” columns we list the number of plots where the respective species contributed to the top 80 % of most abundant spider species.

| **Species** | **Hunting mode** | **Size class** | **Plots** | | |
| --- | --- | --- | --- | --- | --- |
|  |  |  | **Total** | **South** | **North** |
| *Coelotes terrestris* | web builder | l | 29 | 16 | 13 |
| *Walckenaeria corniculans* | web builder | s | 28 | 10 | 18 |
| *Ceratinella brevis* | web builder | s | 25 | 17 | 8 |
| *Pardosa saltans* | hunting | l | 25 | 14 | 11 |
| *Trochosa terricola* | hunting | l | 22 | 7 | 15 |
| *Tenuiphantes flavipes* | web builder | s | 18 | 1 | 17 |
| *Amaurobius fenestralis* | web builder | l | 15 | 13 | 2 |
| *Inermocoelotes inermis* | web builder | l | 14 | 13 | 1 |
| *Hahnia pusilla* | web builder | s | 13 | 2 | 11 |
| *Tapinocyba insecta* | web builder | s | 13 | 0 | 13 |
| *Harpactea lepida* | hunting | l | 12 | 12 | 0 |
| *Diplocephalus picinus* | web builder | s | 11 | 8 | 3 |
| *Dysdera erythrina* | hunting | l | 9 | 0 | 9 |
| *Agroeca brunnea* | web builder | l | 7 | 0 | 7 |
| *Haplodrassus silvestris* | hunting | l | 6 | 0 | 6 |
| *Ozyptila trux* | hunting | l | 6 | 0 | 6 |
| *Hahnia helveola* | web builder | s | 5 | 0 | 5 |
| *Histopona torpida* | web builder | l | 5 | 5 | 0 |
| *Microneta viaria* | web builder | s | 5 | 0 | 5 |
| *Ozyptila praticola* | hunting | s | 5 | 0 | 5 |
| *Diplocephalus latifrons* | web builder | s | 4 | 4 | 0 |
| *Tapinocyba pallens* | web builder | s | 4 | 4 | 0 |
| *Tenuiphantes tenuis* | web builder | s | 4 | 3 | 1 |
| *Walckenaeria cucullata* | web builder | s | 4 | 1 | 3 |
| *Zelotes clivicola* | hunting | l | 4 | 1 | 3 |
| *Callobius claustrarius* | web builder | l | 3 | 3 | 0 |
| *Centromerus dilutus* | web builder | s | 3 | 0 | 3 |
| *Macrargus rufus* | web builder | l | 3 | 0 | 3 |
| *Walckanearia cuspidata* | web builder | s | 3 | 3 | 0 |
| *Walckenaeria dysderoides* | web builder | s | 3 | 1 | 2 |
| *Centromerus brevipalpus* | web builder | s | 2 | 0 | 2 |
| *Haplodrassus soerenseni* | hunting | l | 2 | 0 | 2 |
| *Pelecopsis radicicola* | web builder | s | 2 | 0 | 2 |
| *Tenuiphantes alacris* | web builder | s | 2 | 1 | 1 |
| *Tenuiphantes tenebricola* | web builder | s | 2 | 2 | 0 |
| *Cryphoeca silvicola* | web builder | s | 1 | 1 | 0 |
| *Erigonella hiemalis* | web builder | s | 1 | 1 | 0 |
| *Gonatium rubellum* | web builder | s | 1 | 1 | 0 |
| *Pardosa lugubris* | hunting | l | 1 | 0 | 1 |
| *Piratula hygrophila* | hunting | l | 1 | 0 | 1 |
| *Zelotes subterraneus* | hunting | l | 1 | 0 | 1 |

Additional file 1: Table S2 Ground beetle species contributing to the analyzed top 80 % of most abundant ground beetle species per plot. Species < 100 mg mean individual fresh biomass were categorized as s = small and species ≥ 100 mg were categorized as l = large. In the “plots” columns we list the number of plots where the respective species contributed to the top 80 % of most abundant ground beetle species.

| **Species** | **Feeding type** | **Size class** | **Plots** | | |
| --- | --- | --- | --- | --- | --- |
|  |  |  | **Total** | **South** | **North** |
| *Pterostichus oblongopunctatus* | predator | s | 33 | 15 | 18 |
| *Abax parallelepipedus* | predator | l | 29 | 14 | 15 |
| *Abax ovalis* | predator | s | 25 | 13 | 12 |
| *Pterostichus burmeisteri* | predator | s | 18 | 0 | 18 |
| *Abax parallelus* | predator | l | 12 | 1 | 11 |
| *Carabus problematicus* | predator | l | 10 | 7 | 3 |
| *Notiophilus biguttatus* | predator | s | 10 | 2 | 8 |
| *Pterostichus niger* | predator | l | 8 | 3 | 5 |
| *Carabus auronitens* | predator | l | 7 | 5 | 2 |
| *Harpalus laevipes* | predator | s | 5 | 0 | 5 |
| *Carabus violaceus* | predator | l | 3 | 0 | 3 |
| *Molops piceus* | predator | s | 3 | 3 | 0 |
| *Badister lacertosus* | predator | s | 2 | 0 | 2 |
| *Carabus coriaceus* | predator | l | 2 | 0 | 2 |
| *Carabus glabratus* | predator | l | 2 | 0 | 2 |
| *Carabus nemoralis* | predator | l | 2 | 1 | 1 |
| *Nebria brevicollis* | predator | s | 2 | 0 | 2 |
| *Notiophilus rufipes* | predator | s | 2 | 0 | 2 |
| *Amara ovata* | phytophagy | s | 1 | 1 | 0 |
| *Bembidion lampros* | predator | s | 1 | 0 | 1 |
| *Carabus convexus* | predator | l | 1 | 0 | 1 |
| *Nebria salina* | predator | s | 1 | 0 | 1 |

Additional file 1: Table S3 Model summaries for the spider community metrics versus proportions of conifers (APA Douglas fir & APA Norway spruce) with north-south interaction.

|  |  | Value | Std.Error | DF | t-value | p-value |
| --- | --- | --- | --- | --- | --- | --- |
| *mean* ∆^13^C | (Intercept) | 2.75 | 0.24 | 29 | 11.3 | <0.001 |
|  | North | 0.44 | 0.34 | 6 | 1.3 | 0.243 |
|  | scale(APA Spruce) | -0.58 | 0.24 | 29 | -2.4 | 0.021 |
|  | scale(APA Douglas) | -0.82 | 0.18 | 29 | -4.5 | <0.001 |
|  | North:scale(APA Spruce) | -0.49 | 0.33 | 29 | -1.5 | 0.148 |
| *minimum* ∆^13^C | (Intercept) | 1.64 | 0.15 | 29 | 10.7 | <0.001 |
|  | North | 0.12 | 0.22 | 6 | 0.6 | 0.602 |
|  | scale(APA Spruce) | -0.86 | 0.12 | 29 | -7.1 | <0.001 |
|  | scale(APA Douglas) | -1.01 | 0.16 | 29 | -6.2 | <0.001 |
|  | North:scale(APA Douglas) | 0.51 | 0.22 | 29 | 2.3 | 0.028 |
| *maximum* ∆^13^C | (Intercept) | 3.80 | 0.17 | 29 | 23.0 | <0.001 |
|  | North | -0.05 | 0.23 | 6 | -0.2 | 0.848 |
|  | scale(APA Spruce) | -0.48 | 0.13 | 29 | -3.7 | <0.001 |
|  | scale(APA Douglas) | -0.82 | 0.17 | 29 | -4.7 | <0.001 |
|  | North:scale(APA Douglas) | 0.45 | 0.24 | 29 | 1.9 | 0.065 |
| *range* ∆^13^C | (Intercept) | 2.08 | 0.12 | 31 | 17.0 | <0.001 |
|  | scale(APA Spruce) | 0.31 | 0.12 | 31 | 2.5 | 0.017 |
| *mean* ∆^15^N | (Intercept) | 6.02 | 0.17 | 30 | 35.3 | <0.001 |
|  | North | 0.93 | 0.24 | 6 | 3.9 | 0.008 |
|  | scale(APA Spruce) | 0.20 | 0.11 | 30 | 1.8 | 0.076 |
|  | scale(APA Douglas) | -0.33 | 0.11 | 30 | -3.1 | 0.004 |
| *minimum* ∆^15^N | (Intercept) | 4.39 | 0.17 | 31 | 26.5 | <0.001 |
|  | scale(APA Douglas) | -0.40 | 0.13 | 31 | -3.2 | 0.003 |
| *maximum* ∆^15^N | (Intercept) | 7.72 | 0.24 | 30 | 32.3 | <0.001 |
|  | North | 1.63 | 0.34 | 6 | 4.8 | 0.003 |
|  | scale(APA Spruce) | 0.31 | 0.18 | 30 | 1.8 | 0.087 |
|  | scale(APA Douglas) | -0.29 | 0.18 | 30 | -1.6 | 0.110 |
| *range* ∆^15^N | (Intercept) | 3.46 | 0.25 | 31 | 14.0 | <0.001 |
|  | North | 1.38 | 0.35 | 6 | 4.0 | 0.007 |
|  | scale(APA Spruce) | 0.26 | 0.18 | 31 | 1.4 | 0.159 |
| IRic | (Intercept) | 0.05 | 0.00 | 31 | 11.7 | <0.001 |
|  | scale(APA Spruce) | 0.01 | 0.00 | 31 | 1.8 | 0.083 |
| IDiv | (Intercept) | -0.33 | 0.09 | 32 | -3.5 | 0.001 |
|  | North | -0.29 | 0.13 | 6 | -2.2 | 0.070 |
| IDis | (Intercept) | 0.29 | 0.04 | 32 | 7.7 | <0.001 |
| IEve | (Intercept) | 0.31 | 0.04 | 30 | 8.3 | <0.001 |
|  | North | 0.10 | 0.05 | 6 | 2.0 | 0.096 |
|  | scale(APA Douglas) | -0.07 | 0.04 | 30 | -1.9 | 0.066 |
|  | North:scale(APA Douglas) | 0.15 | 0.05 | 30 | 2.7 | 0.011 |
| IUni | (Intercept) | 0.58 | 0.05 | 32 | 10.9 | <0.001 |
|  | North | -0.15 | 0.08 | 6 | -2.0 | 0.088 |
| SEAc | (Intercept) | 2.37 | 0.16 | 32 | 15.0 | <0.001 |

Additional file 1: Table S4 Model summaries for the spider community metrics versus herb vegetation complexity (Herb complexity), canopy openness, total deadwood volume and neighborhood diversity (NDiv) with north-south interaction.

|  |  | Value | Std.Error | DF | t-value | p-value |
| --- | --- | --- | --- | --- | --- | --- |
| *mean* ∆^13^C | (Intercept) | 3.17 | 0.31 | 29 | 10.1 | <0.001 |
|  | South | -0.14 | 0.47 | 6 | -0.3 | 0.768 |
|  | scale(log(Herb complexity + 1)) | -0.42 | 0.22 | 29 | -1.9 | 0.074 |
|  | scale(log(Total deadwood volume + 1)) | 0.36 | 0.28 | 29 | 1.3 | 0.213 |
|  | South:scale(log(Total deadwood volume + 1)) | -0.75 | 0.44 | 29 | -1.7 | 0.098 |
| *minimum* ∆^13^C | (Intercept) | 1.71 | 0.16 | 31 | 10.6 | <0.001 |
|  | scale(Canopy openness) | -0.49 | 0.16 | 31 | -3.0 | 0.006 |
| *maximum* ∆^13^C | (Intercept) | 3.82 | 0.22 | 29 | 17.4 | <0.001 |
|  | South | 0.17 | 0.33 | 6 | 0.5 | 0.616 |
|  | scale(log(Herb complexity + 1)) | -0.22 | 0.16 | 29 | -1.4 | 0.175 |
|  | scale(log(Total deadwood volume + 1)) | 0.41 | 0.20 | 29 | 2.1 | 0.048 |
|  | South:scale(log(Total deadwood volume + 1)) | -0.72 | 0.31 | 29 | -2.4 | 0.025 |
| *range* ∆^13^C | (Intercept) | 2.08 | 0.12 | 31 | 16.6 | <0.001 |
|  | scale(Canopy openness) | 0.26 | 0.13 | 31 | 2.1 | 0.047 |
| *mean* ∆^15^N | (Intercept) | 6.89 | 0.17 | 30 | 40.4 | <0.001 |
|  | South | -0.81 | 0.25 | 6 | -3.2 | 0.018 |
|  | scale(log(Herb complexity + 1)) | -0.23 | 0.13 | 30 | -1.8 | 0.077 |
|  | scale(Tree neighborhood diversity) | -0.19 | 0.12 | 30 | -1.6 | 0.122 |
| *minimum* ∆^15^N | (Intercept) | 4.39 | 0.15 | 31 | 30.2 | <0.001 |
|  | scale(log(Herb complexity + 1)) | -0.32 | 0.14 | 31 | -2.2 | 0.034 |
| *maximum* ∆^15^N | (Intercept) | 9.45 | 0.26 | 29 | 36.5 | <0.001 |
|  | South | -1.64 | 0.37 | 6 | -4.4 | 0.005 |
|  | scale(log(Herb complexity + 1)) | 0.19 | 0.26 | 29 | 0.7 | 0.476 |
|  | scale(Tree neighborhood diversity) | -0.26 | 0.18 | 29 | -1.4 | 0.161 |
|  | South:scale(log(Herb complexity + 1)) | -0.53 | 0.37 | 29 | -1.4 | 0.164 |
| *range* ∆^15^N | (Intercept) | 4.89 | 0.26 | 29 | 18.8 | <0.001 |
|  | South | -1.36 | 0.37 | 6 | -3.7 | 0.010 |
|  | scale(Canopy openness) | 0.28 | 0.18 | 29 | 1.5 | 0.136 |
|  | scale(Tree neighborhood diversity) | -0.34 | 0.23 | 29 | -1.5 | 0.145 |
|  | South:scale(Tree neighborhood diversity) | 0.52 | 0.37 | 29 | 1.4 | 0.165 |
| IRic | (Intercept) | 0.05 | 0.00 | 31 | 12.6 | <0.001 |
|  | scale(Canopy openness) | 0.01 | 0.00 | 31 | 3.1 | 0.004 |
| IDiv | (Intercept) | -0.56 | 0.10 | 29 | -5.8 | <0.001 |
|  | South | 0.19 | 0.14 | 6 | 1.4 | 0.219 |
|  | scale(Canopy openness) | -0.17 | 0.09 | 29 | -1.9 | 0.068 |
|  | scale(log(Herb complexity + 1)) | 0.12 | 0.08 | 29 | 1.6 | 0.126 |
|  | South:scale(Canopy openness) | 0.24 | 0.14 | 29 | 1.7 | 0.103 |
| IDis | (Intercept) | 0.29 | 0.07 | 26 | 4.3 | <0.001 |
|  | South | 0.11 | 0.09 | 6 | 1.2 | 0.272 |
|  | scale(Canopy openness) | -0.06 | 0.04 | 26 | -1.5 | 0.137 |
|  | scale(log(Herb complexity + 1)) | 0.09 | 0.05 | 26 | 1.8 | 0.083 |
|  | scale(log(Total deadwood volume + 1)) | 0.00 | 0.04 | 26 | 0.1 | 0.949 |
|  | South:scale(Canopy openness) | 0.14 | 0.06 | 26 | 2.3 | 0.030 |
|  | South:scale(log(Herb complexity + 1)) | -0.12 | 0.07 | 26 | -1.8 | 0.080 |
|  | South:scale(log(Total deadwood volume + 1)) | -0.14 | 0.07 | 26 | -2.1 | 0.046 |
| IEve | (Intercept) | 0.42 | 0.04 | 30 | 10.5 | <0.001 |
|  | South | -0.13 | 0.06 | 6 | -2.2 | 0.069 |
|  | scale(Tree neighborhood diversity) | 0.00 | 0.04 | 30 | -0.1 | 0.908 |
|  | South:scale(Tree neighborhood diversity) | -0.09 | 0.06 | 30 | -1.5 | 0.149 |
| IUni | (Intercept) | 0.41 | 0.05 | 26 | 7.7 | <0.001 |
|  | South | 0.13 | 0.08 | 6 | 1.6 | 0.152 |
|  | scale(Canopy openness) | 0.07 | 0.05 | 26 | 1.3 | 0.198 |
|  | scale(log(Herb complexity + 1)) | -0.08 | 0.06 | 26 | -1.3 | 0.204 |
|  | scale(Tree neighborhood diversity) | -0.09 | 0.05 | 26 | -2.0 | 0.051 |
|  | South:scale(Canopy openness) | -0.10 | 0.08 | 26 | -1.3 | 0.198 |
|  | South:scale(log(Herb complexity + 1)) | 0.20 | 0.08 | 26 | 2.5 | 0.018 |
|  | South:scale(Tree neighborhood diversity) | 0.14 | 0.08 | 26 | 1.8 | 0.080 |
| SEAc | (Intercept) | 2.37 | 0.15 | 31 | 15.9 | <0.001 |
|  | scale(Canopy openness) | 0.37 | 0.15 | 31 | 2.4 | 0.020 |

Additional file 1: Table S5 Model summaries for the ground beetle community metrics versus proportions of conifers (APA Douglas fir & APA Norway spruce) with north-south interaction.

|  |  | **Value** | **Std.Error** | **DF** | **t-value** | **p-value** |
| --- | --- | --- | --- | --- | --- | --- |
| *mean* ∆^13^C | (Intercept) | 2.29 | 0.10 | 30 | 22.7 | <0.001 |
|  | scale(APA Spruce) | -0.76 | 0.11 | 30 | -7.2 | <0.001 |
|  | scale(APA Douglas) | -0.75 | 0.11 | 30 | -7.2 | <0.001 |
| *minimum* ∆^13^C | (Intercept) | 1.24 | 0.20 | 30 | 6.3 | <0.001 |
|  | scale(APA Spruce) | -1.12 | 0.14 | 30 | -7.8 | <0.001 |
|  | scale(APA Douglas) | -1.05 | 0.14 | 30 | -7.3 | <0.001 |
| *maximum* ∆^13^C | (Intercept) | 3.20 | 0.10 | 30 | 31.1 | <0.001 |
|  | scale(APA Spruce) | -0.51 | 0.12 | 30 | -4.4 | <0.001 |
|  | scale(APA Douglas) | -0.65 | 0.12 | 30 | -5.6 | <0.001 |
| *range* ∆^13^C | (Intercept) | 1.97 | 0.20 | 30 | 9.6 | <0.001 |
|  | scale(APA Spruce) | 0.61 | 0.15 | 30 | 4.0 | <0.001 |
|  | scale(APA Douglas) | 0.40 | 0.15 | 30 | 2.6 | 0.013 |
| *mean* ∆^15^N | (Intercept) | 5.04 | 0.16 | 31 | 31.4 | <0.001 |
|  | scale(APA Douglas) | -0.40 | 0.15 | 31 | -2.7 | 0.010 |
| *minimum* ∆^15^N | (Intercept) | 3.96 | 0.40 | 30 | 9.8 | <0.001 |
|  | North | -0.96 | 0.57 | 6 | -1.7 | 0.144 |
|  | scale(APA Spruce) | -0.57 | 0.20 | 30 | -2.9 | 0.007 |
|  | scale(APA Douglas) | -0.61 | 0.20 | 30 | -3.1 | 0.004 |
| *maximum* ∆^15^N | (Intercept) | 6.66 | 0.30 | 32 | 22.3 | <0.001 |
| *range* ∆^15^N | (Intercept) | 0.67 | 0.16 | 31 | 4.1 | <0.001 |
|  | North | 0.58 | 0.23 | 6 | 2.5 | 0.048 |
|  | scale(APA Spruce) | 0.17 | 0.08 | 31 | 2.1 | 0.047 |
| IRic | (Intercept) | -4.99 | 0.38 | 28 | -13.1 | <0.001 |
|  | North | 1.22 | 0.54 | 6 | 2.3 | 0.065 |
|  | scale(APA Spruce) | 1.19 | 0.24 | 28 | 4.9 | <0.001 |
|  | scale(APA Douglas) | 1.04 | 0.24 | 28 | 4.2 | <0.001 |
|  | North:scale(APA Spruce) | -0.54 | 0.35 | 28 | -1.5 | 0.139 |
|  | North:scale(APA Douglas) | -0.83 | 0.35 | 28 | -2.4 | 0.026 |
| IDiv | (Intercept) | 0.78 | 0.02 | 31 | 35.4 | <0.001 |
|  | scale(APA Douglas) | -0.06 | 0.02 | 31 | -3.4 | 0.002 |
| IDis | (Intercept) | 0.58 | 0.03 | 32 | 17.6 | <0.001 |
| IEve | (Intercept) | 0.63 | 0.03 | 32 | 21.1 | <0.001 |
| IUni | (Intercept) | 0.63 | 0.03 | 31 | 20.6 | <0.001 |
|  | scale(APA Douglas) | -0.06 | 0.03 | 31 | -2.0 | 0.052 |

Additional file 1: Table S6 Model summaries for the ground beetle community metrics versus herb vegetation complexity (Herb complexity), canopy openness, total deadwood volume and neighborhood diversity (NDiv) with north-south interaction.

|  |  | **Value** | **Std.Error** | **DF** | **t-value** | **p-value** |
| --- | --- | --- | --- | --- | --- | --- |
| *mean* ∆^13^C | (Intercept) | 2.41 | 0.26 | 28 | 9.5 | <0.001 |
|  | North | -0.30 | 0.36 | 6 | -0.8 | 0.434 |
|  | scale(log(Herb complexity + 1)) | 0.00 | 0.24 | 28 | 0.0 | 0.994 |
|  | scale(log(Total deadwood volume + 1)) | -0.28 | 0.26 | 28 | -1.1 | 0.281 |
|  | North:scale(log(Herb complexity + 1)) | -0.55 | 0.33 | 28 | -1.7 | 0.109 |
|  | North:scale(log(Total deadwood volume + 1)) | 0.49 | 0.34 | 28 | 1.5 | 0.157 |
| *minimum* ∆^13^C | (Intercept) | 1.24 | 0.26 | 31 | 4.7 | <0.001 |
|  | scale(Canopy openness) | -0.53 | 0.22 | 31 | -2.4 | 0.023 |
| *maximum* ∆^13^C | (Intercept) | 3.45 | 0.22 | 29 | 15.8 | <0.001 |
|  | North | -0.34 | 0.32 | 6 | -1.1 | 0.327 |
|  | scale(log(Herb complexity + 1)) | -0.28 | 0.15 | 29 | -1.8 | 0.077 |
|  | scale(log(Total deadwood volume + 1)) | -0.34 | 0.23 | 29 | -1.5 | 0.152 |
|  | North:scale(log(Total deadwood volume + 1)) | 0.45 | 0.30 | 29 | 1.5 | 0.144 |
| *range* ∆^13^C | (Intercept) | 1.97 | 0.24 | 31 | 8.3 | <0.001 |
|  | scale(Canopy openness) | 0.35 | 0.16 | 31 | 2.2 | 0.036 |
| *mean* ∆^15^N | (Intercept) | 5.04 | 0.14 | 30 | 35.8 | <0.001 |
|  | scale(Canopy openness) | -0.34 | 0.14 | 30 | -2.4 | 0.025 |
|  | scale(Tree neighborhood diversity) | -0.41 | 0.14 | 30 | -2.9 | 0.007 |
| *minimum* ∆^15^N | (Intercept) | 3.48 | 0.32 | 30 | 10.9 | <0.001 |
|  | scale(Canopy openness) | -0.59 | 0.19 | 30 | -3.0 | 0.005 |
|  | scale(Tree neighborhood diversity) | -0.45 | 0.19 | 30 | -2.4 | 0.024 |
| *maximum* ∆^15^N | (Intercept) | 6.39 | 0.42 | 28 | 15.1 | <0.001 |
|  | North | 1.08 | 0.61 | 6 | 1.8 | 0.124 |
|  | scale(Canopy openness) | 0.08 | 0.44 | 28 | 0.2 | 0.858 |
|  | scale(log(Herb complexity + 1)) | -0.35 | 0.44 | 28 | -0.8 | 0.440 |
|  | North:scale(Canopy openness) | -1.29 | 0.61 | 28 | -2.1 | 0.043 |
|  | North:scale(log(Herb complexity + 1)) | 1.20 | 0.65 | 28 | 1.8 | 0.077 |
| *range* ∆^15^N | (Intercept) | 0.73 | 0.15 | 28 | 4.9 | <0.001 |
|  | North | 0.63 | 0.22 | 6 | 2.9 | 0.026 |
|  | scale(Canopy openness) | 0.22 | 0.14 | 28 | 1.6 | 0.132 |
|  | scale(log(Herb complexity + 1)) | -0.11 | 0.14 | 28 | -0.8 | 0.431 |
|  | North:scale(Canopy openness) | -0.38 | 0.19 | 28 | -2.0 | 0.058 |
|  | North:scale(log(Herb complexity + 1)) | 0.42 | 0.21 | 28 | 2.0 | 0.052 |
| IRic | (Intercept) | -4.98 | 0.43 | 26 | -11.5 | <0.001 |
|  | North | 1.31 | 0.61 | 6 | 2.1 | 0.078 |
|  | scale(Canopy openness) | 0.71 | 0.35 | 26 | 2.1 | 0.050 |
|  | scale(log(Herb complexity + 1)) | -0.28 | 0.35 | 26 | -0.8 | 0.436 |
|  | scale(log(Total deadwood volume + 1)) | 0.51 | 0.39 | 26 | 1.3 | 0.197 |
|  | North:scale(Canopy openness) | -0.97 | 0.47 | 26 | -2.1 | 0.050 |
|  | North:scale(log(Herb complexity + 1)) | 0.83 | 0.51 | 26 | 1.6 | 0.117 |
|  | North:scale(log(Total deadwood volume + 1)) | -0.85 | 0.49 | 26 | -1.7 | 0.096 |
| IDiv | (Intercept) | 0.78 | 0.02 | 32 | 37.0 | <0.001 |
| IDis | (Intercept) | 0.58 | 0.03 | 32 | 17.6 | <0.001 |
| IEve | (Intercept) | 0.63 | 0.03 | 31 | 21.5 | <0.001 |
|  | scale(log(Total deadwood volume + 1)) | 0.05 | 0.03 | 31 | 1.6 | 0.123 |
| IUni | (Intercept) | 0.63 | 0.03 | 32 | 20.0 | <0.001 |
